# *Stenotrophomonas maltophilia* affects the gene expression profiles of the major pathogens *Pseudomonas aeruginosa* and *Staphylococcus aureus* in an *in vitro* multispecies biofilm model

**DOI:** 10.1128/spectrum.00859-23

**Published:** 2023-10-11

**Authors:** Ifey Alio, Raphael Moll, Tim Hoffmann, Uwe Mamat, Ulrich E. Schaible, Kai Pappenfort, Malik Alawi, Marcel Schie, Roland Thünauer, Johanna Stamm, Holger Rohde, Wolfgang R. Streit

**Affiliations:** 1 Department of Microbiology and Biotechnology, University Hamburg, Hamburg, Germany; 2 Cellular Microbiology, Priority Research Area Infections, Research Center Borstel, Leibniz Lung Center,Leibniz Research Alliance Infection , Borstel Gemany, Borstel, Germany; 3 Institute of Microbiology, Friedrich Schiller University of Jena, Jena, Germany; 4 Bioinformatics Core, UKE Hamburg, Hamburg, Germany; 5 LIV, Leibniz Institute of Experimental Virology, Hamburg, Germany; 6 Institute for Medical Microbiology, Virology and Hygiene, UKE, Eppendorf, Hamburg, Germany; Forschungszentrum Jülich GmbH, Juelich, Germany

**Keywords:** mixed species biofilms, transcriptome analysis, pathogens

## Abstract

*Stenotrophomonas maltophilia* is an emerging lung pathogen. In its natural habitat, it lives together with *Pseudomonas aeruginosa*, *Staphylococcus aureus*, and other pathogens. Here, we provide the first evidence that *S. maltophilia* interferes with the metabolism and physiology of other species when co-cultivated in dual- and triple-species biofilms. CSLM analyses implied that *S. maltophilia* was in general the first to colonize the bottom layer in multispecies biofilms. Structural patterns and niche formation resulting in distinct layer formation within the biofilm were observed on a species- and strain-dependent level for *S. maltophilia*. Furthermore, gene expression profiles of *S. aureus* and *P. aeruginosa* were strongly affected by the presence of *S. maltophilia*. The *S. maltophilia* metabolism was mostly fermentative in multispecies biofilms with varying sets of cytochromes used for anaerobic respiration. One of the most striking observations was that *S. maltophilia* interfered with *P. aeruginosa* LasI-dependent expression of quorum sensing-regulated pathogenicity factors in multispecies biofilms.

**IMPORTANCE:**

In the past, studies have focused on bacterial pathogenicity in mono-species infections, in part ignoring the clinical relevance of diseases caused by more than one pathogen (i.e., polymicrobial infections). However, it is now common knowledge that multiple bacteria species are often involved in the course of an infection. For treatment of such infections, it is absolutely important to understand the dynamics of species interactions at possible infection sites and the molecular mechanisms behind these interactions. Here, we studied the impact of *Stenotrophomonas maltophilia* on its commensals *Pseudomonas aeruginosa* and *Staphylococcus aureus* in multispecies biofilms. We analyzed the 3D structural architectures of dual- and triple-species biofilms, niche formation within the biofilms, and the interspecies interactions on a molecular level. RNAseq data identified key genes involved in multispecies biofilm formation and interaction as potential drug targets for the clinical combat of multispecies infection with these major pathogens.

## INTRODUCTION

Assembly of surface adherent multicellular communities is a key phenotype of apparently all microorganisms. These biofilms essentially support survival of microorganisms in their native habitats (1 – 4). Over the last decades, the molecular mechanisms driving bacterial biofilm formation have been untraveled in great detail. However, while most studies focused on single-species cultures, significant evidence demonstrates that bacteria biofilm formation involves multiple, cross-kingdom species (5 – 7). This essentially holds true also for medically important biofilms associated with invasive human diseases, e.g., pulmonary or skin infections immunocompromised patients (8 – 10). In these scenarios, infections are caused by multiple Gram-negative and Gram-positive bacteria, as well as fungi, being co-localized in complex biofilms (11 – 13). For example, lung infection in cystic fibrosis patients is usually caused by bacteria such as *Pseudomonas aeruginosa*, *Stenotrophomonas maltophilia*, and *Staphylococcus aureus*, as well as *Aspergillus fumigatus* and additional fungi (14, 15). Clearly, these pathogens are well equipped to cause invasion and disease by expressing a plethora of pathogenicity factors, including biofilm formation, fostering their ability to persist in hostile host environments (16 – 19).

Only recently, however, evidence became available describing interactions of medically relevant pathogens in dual-species biofilms. These biofilms include beneficial as well as harmful interactions. Few of these have used combinations of *P. aeruginosa* with *S. aureus*. It has been shown for instance that *P. aeruginosa* can enhance the efficacy of norfloxacin against *S. aureus* in a dual-species biofilm (20). Another study demonstrated that the supernatant of *P. aeruginosa* cultures can decrease the susceptibility of *S. aureus* toward vancomycin (20, 21). Additionally, it was shown that 3-oxo-C12 homoserine lactone, a cell-cell signaling molecule of *P. aeruginosa*, affects the morphology of *C. albicans*, toward less filamentation in co-cultures (22).

In a multispecies biofilm, the dynamics of interspecies interactions such as communication, competition for nutrients and resources as well as a shared resilience toward environmental stress such as antimicrobial treatment can shape the overall physiology and function of the biofilm (23 – 29).

This synergistic and protective effect of mixed-species biofilms toward antimicrobials makes treatment, therefore, more challenging in clinical settings.

Recently, we showed that the 3D structures of single-species *S. maltophilia* biofilm strains strongly varied on strain-specific level (19).

Within this framework, we were interested to study the influence of *S. maltophilia* on niche formation, quorum sensing, and nutrient uptake in other pathogens such as *P. aeruginosa* and *S. aureus* in multispecies laboratory-grown biofilms. Therefore, in this study, we established multispecies biofilms with either two or three species using the above-named model pathogens. In addition, we included *Candida albicans*, a fungi that is also frequently observed in lung infections (30 – 32).

To achieve this, we first chromosomally tagged all bacterial strains with a wide range of fluorescent reporter genes [green (sGFP), blue (TagBFP), cyan (AmCyan), cerulean, (mCerulean), red (mCherry and tdTomato), orange (mOrange), and yellow (eYFP)] to follow multispecies population dynamics and niche formation. Additional deep RNAseq analyses of dual- and triple-species biofilms in combination with confocal imaging using key metabolic reporter genes as markers gave first and fascinating insights into the complex interaction, niche formation, and competition of these major pathogens on a molecular level.

## MATERIALS AND METHODS

### Bacterial strains, chemicals, and growth conditions

Strains and plasmids used in this study are summarized in Table 1. All strains were cultured in either Luria-Bertani (LB) medium (10 g/L tryptone, 5 g/L yeast extract, and 5 g/L NaCl) or 10% LB medium at 28°C or 37°C.

**TABLE 1:** Bacterial strains used and plasmids

Strains and plasmids	Description	Reference/source
*E. coli* DH5α	F- ɸ80dlacZΔM15 Δ(argF-lacZYA) U169 endA1 hsdR17 (rK-, mK-) supE44 thi-1 recA1 gyrA96 relA1	(33)
*E. coli* SM10ƛpir	thi thr leu tonA lacY supE recA::RP4-2-Tc::Mu Km λpir	
*P. aeruginosa* PAO1	Wild-type isolate	(34)
PAO1_sfGFP_UHH07	Mini Tn7T, pc promotor, Gm^r^, sfGFP	This study
PAO1_mCherry_UHH08	Mini Tn7T, pc promotor, Gm^r^, mCherry	This study
PAO1_eYFP_UHH09	Mini Tn7T, pc promotor, Gm^r^, eYFP	This study
PAO1_mOrange_UHH10	Mini Tn7T, pc promotor, Gm^r^, mOrange	This study
*S. maltophilia* K279a	Wild-type clinical reference isolate	(35)
*S. maltophilia* SM454	Wild-type clinical isolate	(19)
K279a_sfGFP_UHH01	Mini Tn7T, pc promotor, Gm^r^, sfGFP	This study
K279a_tdtomato_UHH02	Mini Tn7T, pc promotor, Gm^r^, tdtomato	This study
K279a_mOrange_UHH03	Mini Tn7T, pc promotor, Gm^r^, mOrange	This study
K279a_eYFP_UHH04	Mini Tn7T, pc promotor, Gm^r^, eYFP	This study
K279a_mCerulean-UHH05	Mini Tn7T, pc promotor, Gm^r^, mCerulean	This study
K279a_BFP_UHH06	Mini Tn7T, pc promotor, Gm^r^, tagBFP	This study
*S. aureus* SH1000	Wild-type reference isolate	(36)
SH1000_sfGFP_UKE01	PCM29-SarA–sfGFP	This study
SH1000_AmCyan_UKE02	PCM29-SarA–AmCyan	This study
SH1000_mCherry_UKE03	PCM29-SarA–mCherry	This study
*Candida albicans* SC5314	Wild-type isolate	Provided by A. Gacser, Hungary
SC5314_sfGFP_UHH11	Mutant expressing EGFP	A. Gacser, Hungary
Plasmids		
pRK2013	KanR; RK2-derived helper plasmid carrying the tra and mob genes for mobilization of plasmids containing oriT	(37)
pUX-BF13	Transmissible plasmid containing oriVR6K. It carries tns{A, B,C,D,E} genes, which are necessary for miniTn7 transfer	(38)
pUC18T-miniTn7T-Gm^r^	FRT-flanked Gm^r^ marker was inserted within miniTn7 element, Amp^r^	(39)
pUC18T-miniTn7T-Gm^r^-Pc	Constitutive pc promotor of the class III Integron of *Delftia acidovorans* inserted after the Gm^r^ cassette	This study
pUC18T-miniTn7T-Gm^r^-Pc-sfGFP	Fluorescent gene s*fGFP* inserted after the pc promotor	This study
pUC18T-miniTn7T-Gm^r^-Pc-mCherry	Fluorescent gene *mCherry* inserted after the pc promotor	This study
pUC18T-miniTn7T-Gm^r^-Pc-tdtomato	Fluorescent gene *td tomato* inserted after the pc promotor	This study
pUC18T-miniTn7T-Gm^r^-Pc-cyan	Fluorescent gene *eCyan* inserted after the pc promotor	This study
pUC18T-miniTn7T-Gm^r^-Pc-tagBFP	Fluorescent gene *tagBFP* inserted after the pc promotor	This study
pUC18T-miniTn7T-Gm^r^-Pc-mCerulean	Fluorescent gene *mCerulean* inserted after the pc promotor	This study
pUC18T-miniTn7T-Gm^r^-Pc-mOrange	Fluorescent gene *mOrange* inserted after the pc promotor	This study
pUC18T-miniTn7T-Gm^r^-Pc-eYFP	Fluorescent gene eyfp inserted after the pc promotor	This study
pCM29_SarA	Shuttle vector for *S. aureus*, expression of fluorescent genes from the SarA-P1 promoter; carries chloramphenicol resistance Cm^r^, only functional in Gram positives	This study
pCM29_SarA_sfGFP	Fluorescent gene *sfGFP* inserted after the SarA promotor	This study
pJ-mCherry	Fluorescent gene *mCherry* inserted after the SarA promotor	This study
pJ-AmCyan	Fluorescent gene *AmCyan* inserted after the SarA promotor	This study
pBBR1-MCS	Broad host range vector, low copy, Cm^r^	(40)
pBBR1-MCS_P1360-sfGFP-P4401-mCerulean	pBBR1-MCS with the promotor fusion *cyoA* (smlt1360) promotor fused to *sfGFP* and the promotor cyoA (smlt4401) fused to *mCerulean*	This study

### Molecular cloning and labeling bacterial strains with fluorescent reporter genes

Most of the strains used in this study were chromosomally tagged with a fluorescence protein via the mini Tn7T transposon according to previously published protocols (39, 41) and as recently published by some of the authors on this manuscript (42). Mamat et al. (42) demonstrated that the Tn7-tagged variants are not affected in their fitness, growth, and biofilm formation.

*S. aureus* SH1000 was labeled with the plasmid pCM29. The different plasmids used are listed in Table 1. Plasmid pCM29 (43) is a widely used plasmid providing constitutive expression of sfGFP. To extend multi-color labeling abilities, additional plasmids for in *trans* expression of mCherry and AmCyan in *S. aureus* were constructed. To this end, codon optimized mCherry- and AmCyan-encoding genes were synthesized (Eurofins, Ebersberg, Germany) according to previously published sequences (44). Sub-cloning into pCM29 was achieved via a Gibson cloning approach using primers pCM_sarA_fwd and pCM-sarA_rev for backbone amplification and primer pairs mCherry_für_sarA_fwd with mCherry_rev and AmCyan_für_sarA_fwd with AmCyan_rev, respectively. The resulting constructs referred to as pJ-mCherry and pJ-AmCyan, which carry fluorophore-encoding sequences, are under the control of the *sarA* promoter resulting in constitutive fluorophore production. Correctness was verified by sequencing. Plasmids pJ-mCherry, pJ-AmCyan, and pCM29 were introduced into *S. aureus* SH1000 via electroporation according to the protocols of Grosser and Richardson (45).

A *C. albicans* strain labeled with green fluorescent protein (GFP) was provided by Prof. A. Gacser, Department of Microbiology, University of Szeged.

### Cultivation of biofilms in flow chambers or µ-slides

In order to investigate the biofilm architecture, strains were cultivated in three-channel flow chambers (31) or µ-slide eight-well (ibiTreat, catalog no. 80826, ibidi USA, Inc., Fitchburg, Wisconsin). In flow chambers, biofilms were grown according to the methods described in reference (19). For inoculation, an overnight culture of the various strains was adjusted to 4.0 × 10^7^ CFU/mL in 10% LB medium. The cell ratios were 1:1 for our dual- and triple-species biofilms. The µ-slides were inoculated with 350 µL of the diluted culture per well and incubated for 24 h, 48 h, and 72 h at 37°C. The flow chambers were inoculated with 300 µL of the diluted culture per channel and incubated at 28°C for 1 h to allow the cells to adhere, followed by resumption of the flow at a rate of 50 µL/minute. All experiments were performed at 37°C or 28°C with 10% LB medium.

### Fluorescence imaging analysis of biofilms

Visualization of flow chamber and µ-slide biofilms was performed using a confocal laser scanning microscope (CLSM) Axio Observer.Z1/7 LSM 800 with Airyscan (Carl Zeiss Microscopy GmbH, Jena, Germany) and a C-Apochromat 63×/1.20 W Korr UV VisIR objective. The microscope settings for the different fluorescent dyes are shown in Table S3. The analysis of the CLSM images and three-dimensional reconstructions were done with the ZEN software (version 2.3, Carl Zeiss Microscopy GmbH, Jena, Germany). Biofilm architecture were analyzed at least at three different positions for each strain, and one representative CLSM image was chosen.

#### Image analysis

The fluorescent images are analyzed using an algorithm written in Python 3 Reference Manual: Guide books (acm.org) (46). First, the crosstalk for each combination of fluorescent dyes is determined by calculating the distribution of pixel-to-pixel intensity bleed-through $I_{zyx}^{\text{unpopulated}}/I_{zyx}^{populated}$ between the channels. For each channel, the maximum of this distribution is taken as the crosstalk coefficient. Second, each biofilm image is subsequently corrected for the previously calculated crosstalk. This is followed by a thresholding procedure comprising a Gaussian blur and automated Li thresholding (47, 48). Finally, the thresholder voxel volumes are summed up for each z-slice of the image as well as in total and are normalized to the total volume of a z-slice or biofilm, respectively. For each combination of bacterial species and for each time (24 h, 48 h, and 72 h post infection), the volumes—per slice and in total—are averaged over a set of three different samples (different positions) and are presented with the corresponding standard deviation (https://github.com/MarcelSchie/Biofilms).

### RNAseq and data analysis

For RNA preparation, biofilms were cultivated at 37°C for 72 h under static conditions using 10% LB medium. Antibiotics were added if possible to the media of overnight cultures to avoid contaminations. Biofilms were grown in six-well plates (4 mL/well) for 72 h. After that, the supernatant was discarded, and the biofilms were resuspended in 2 mL of 20% stop mix (95% ethanol and 5% phenol) and pelleted for 20 minutes at 4°C. Then, the pellets were washed three times with phosphate buffered saline (PBS) and were frozen in liquid nitrogen for later analysis. For each sample, three biological replicates were prepared. The pellets were sent to Vertis Biotechnologie AG, Freising, Germany. The company conducted the next steps of RNAseq. The next generation sequencing (NGS) libraries were single-read sequenced on an Illumina NextSeq 500 system using 75-bp-read length. The NGS library pool was analyzed on a Shimadzu MultiNA microchip electrophoresis system. For detailed information, see the data delivery notes of all samples in Table S6.

Sequence reads were processed with fastp (v0.20.1) (49) to remove sequences originating from sequencing adapters and sequences of low quality (Phred quality score below 15) from the 3′ end of the sequence reads (FASTP). Reads were then aligned to the yeast RefSeq assembly GCF_000182965.3 with STAR (v2.7.9a) (50). Only unaligned reads, which were obtained using the STAR option “--outReadsUnmapped,” were then aligned to the corresponding bacterial reference assemblies (GCF_000072485.1, GCF_000006765.1, and GCF_000013425.1, respectively) with the Burrows Wheeler Aligner (51). Counts of reads per gene were obtained using feature counts (52). Differential expression was assessed with DESeq2 (v1.34.0) (53). A gene was considered significantly differentially expressed if the corresponding absolute log2-transformed fold change was not less than 2, and the false discovery rate did not exceed a value of 0.1.

### Lactate measurement of biofilm supernatants

Biofilms were grown in LB 10% at a start OD600 of 0.05 (single-species biofilms) or 0.1 (multispecies biofilms) for 72 h at 37°C under static conditions. Subsequently, the biofilm supernatants were collected and sterilized via 0.2-µm filters from clear line. Afterwards, the lactate amount in the supernatants was determined with the D-Lactic acid/L-lactic acid kit from Boehringer Mannheim/R-BIOPHARM. The measurement was performed according to the manufacturer’s protocol.

## RESULTS

We recently demonstrated that the emerging pathogen *S. maltophilia* forms thick biofilms that are highly diverse on a strain-specific level and with respect to their 3D structures and gene expression profiles. We further demonstrated that there is a strong variation in genotype and phenotypic traits among *S. maltophilia* clinical isolates (19, 54). Within this setting, we asked to which extent the clinical isolates *S. maltophilia* K279a and SM454 would affect biofilm formation and structure of other pathogens and vice versa. For this, we used *P. aeruginosa* PAO1, *S. aureus* SH1000, and the yeast *C. albicans* SC5314 (Table 1) and cultivated them with SM454 and K279a under biofilm conditions. These strains are all model reference strains used in laboratory research with established complete genomes and are, therefore, ideal for the establishment of our mixed-species biofilm models.

### Image analysis of dual- and triple-species biofilms identifies distinct structural patterns and layer formation

**Fig 1:**
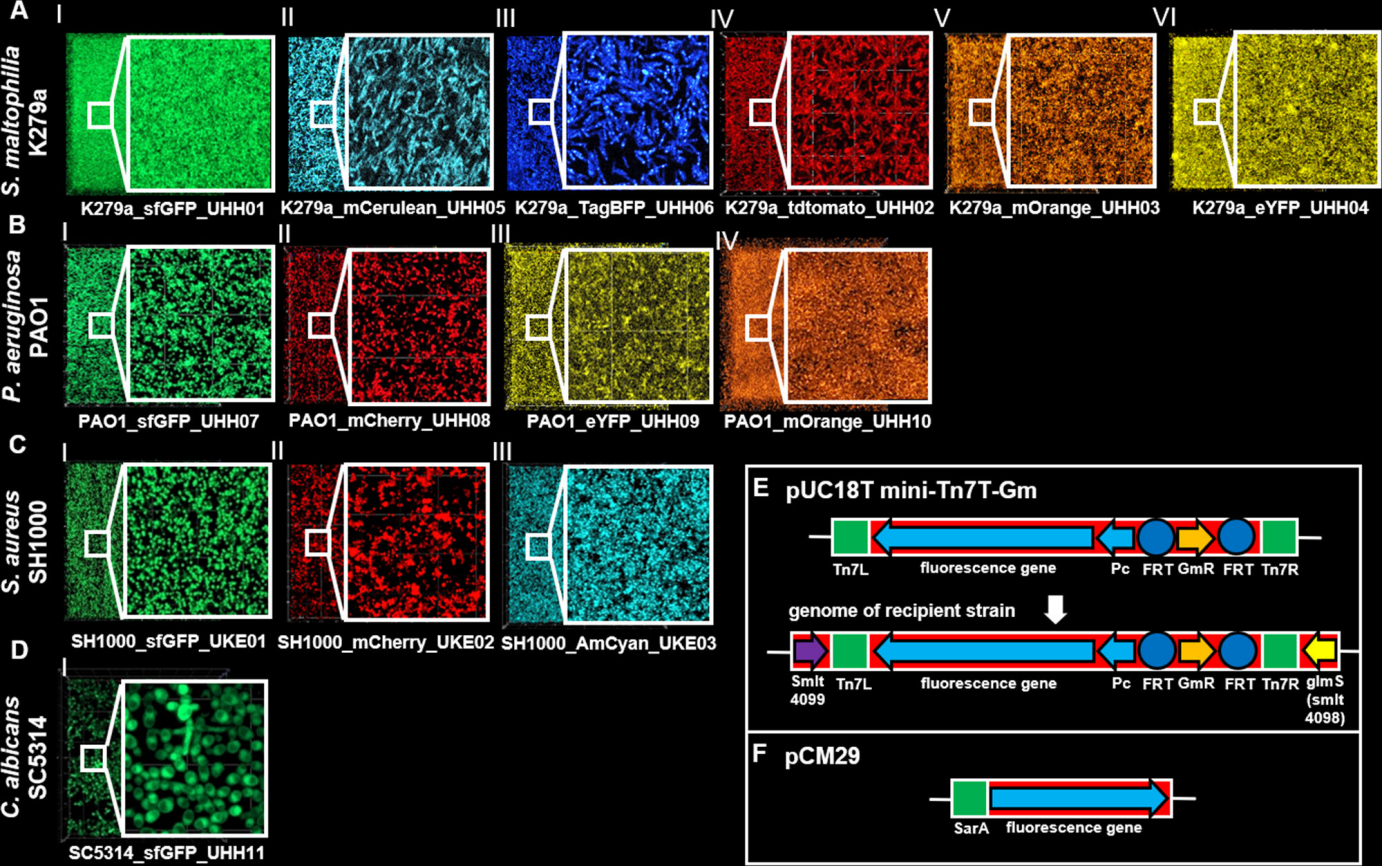
Laser scanning microscope images of fluorescent-labeled species used in this study. The different species *S. maltophilia* and *P. aeruginosa* were labeled with the mini Tn7T transposon. The strain *S. aureus* SH1000 was labeled with the plasmid PCM29 coding the fluorescent proteins under the SarA promotor. *C. albicans* SC5314 was labeled by integrating the fluorescent protein into the chromosome. Panel A shows images of *S. maltophilia* labeled with different fluorescent proteins. Panel B shows *P. aeruginosa* PAO1 labeled with different fluorescent proteins. Panel C shows images of *S. aureus* SH1000 labeled with different fluorescent proteins. Panel D shows *C. albicans* SC5314 labeled with different fluorescent proteins. In panel E, the principle of the Mini-Tn7 transposons for site-specific tagging of bacteria with fluorescent proteins is demonstrated. Panel F shows a part of the plasmid PCM29 encoding the fluorescent proteins under the SarA promotor. This vector was used for tagging the strain *S. aureus* SH1000.

As first step, we tagged the various microorganism with fluorescent reporter genes either inserted into the chromosome or plasmid borne and as described in Materials and Methods (Table 1). Thereby, we produced nine different green fluorescent variants of K279a and SM454 (e.g., green, sfGFP; blue, TagBFP; cyan, AmCyan; cerulean, mCerulean; red, mCherry and tdTomato; orange, mOrange; and yellow, eYFP). Similarly, we obtained chromosomally tagged green, red, orange, and yellow variants of the opportunistic pathogen *P. aeruginosa* PAO1 (Table 1). The fluorescent reporter genes in *S. maltophilia and P. aeruginosa* were inserted 3′ end of the glucosamine phosphate synthase gene (*glmS*) of the different organisms. The correct positions of the fluorescent reporter genes were verified by PCR and using specific primers. All strains were verified for their equally strong fluorescence signal under CLSM (Fig. 1) and were not altered in their fitness or other phenotypic traits such as virulence and biofilm formation (42).

The *S. maltophilia* strains K279a and SM454 were co-cultivated with the tagged variants of *P. aeruginosa* PAO1, *S. aureus* SH1000 and the yeast strain *C. albicans* SC5314.

**Fig 2:**
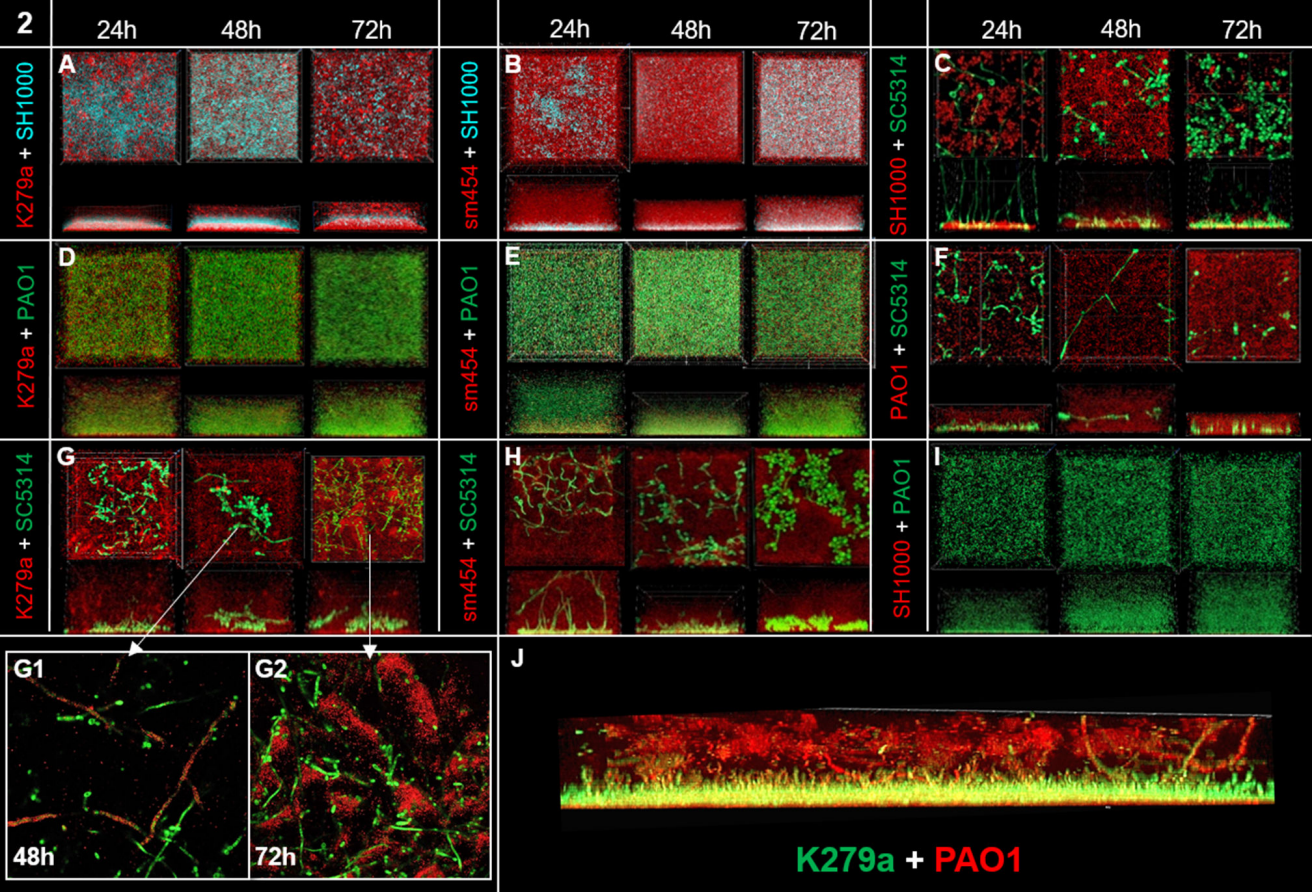
Dual-species biofilms confocal analyses and layer formation. Dual-species biofilms were grown under static conditions at 37°C in 10% LB and analyzed with confocal microscopy. Images were taken after 24 h, 48 h, and 72 h. Dual-species biofilm of *S. maltophilia* K279a tdTomato (red) + *S. aureus* SH1000 eCyan (cyan) (**A**), *S. maltophilia* K279a tdTomato (red) + *P. aeruginosa* PA01 GFP (green) (**B**), *S. maltophilia* K279a tdTomato (red) + *C. albicans* SC5314 sfGFP (green) (**C**), *S. aureus* SH1000 mCherry (red) + *C. albicans* SC5314 sfGFP (green) (**D**), *P. aeruginosa* PA01 mCherry (red) + *C. albicans* SC5314 sfGFP (green) (**E**), and *P. aeruginosa* PA01 GFP (green) + *S. aureus* SH1000 mCherry (red) (**F**). In (C1 + 2), the attachment of *S. maltophilia* K279a tdTomato (red) on the hyphae of *C. albicans* SC5314 sfGFP (green) is shown after 48 h and 72 h. (**G**) Dual-species biofilm of *S. maltophilia* K279a sfGFP (green) and *P. aeruginosa* mCherry (red) grown under flow conditions for 72 h at 28°C. Image analysis shows a more pronounced layer formation. .

For the dual-species biofilms, we analyzed a total of nine combinations, and for the triple-species biofilms, we analyzed three different combinations. The main findings for the various combinations are summarized in Table 2 and below. While we were able to grow all species in mixed-species biofilms in a ratio of 1:1, it became evident that SH1000 was in general outgrown and almost not detectable in the combination with PAO1 (Fig. 2; Table 2).

**TABLE 2:** Main phenotypic traits and key findings observed in multispecies biofilms of major wound and lung pathogens after 24, 48, and 72 h growth at 37°C

	Key findings from CSL image analysis	Cell ratio (%)
Dual species		
*S. maltophilia* K279a + *S. aureus* SH1000	•Distinct layer formation •Triple layer sandwich-like structures were most pronounced with K279a mainly colonizing the bottom and upper layers	24 h: 76/24 48 h: 58/42 72 h: 75/25
*S. maltophilia* K279a + *P. aeruginosa* PAO1	•Layer formation with K279a forming the first bottom layer •PAO1 is predominantly in the upper layers •PAO1 cells dominated the biofilm over time	24 h: 24/76 48 h: 30/70 72 h: 7/93
*S. maltophilia* K279a + *C. albicans* SC5314	•K279a and *C. albicans* on bottom layers •Long hypha formation of *C. albicans* after 24 h and 48 h •K279a cells directly attach to the hyphae of *C. albicans* •Sm454 does not attach to hyphae	24 h: 34.5/65.5 48 h: 32.6/67.4 72 h: 38.3/61.7
*S. aureus* SH1000 + *C. albicans* SC5314	•*S. aureus* and *C. albicans* on bottom •Hypha formation of *C. albicans* in early biofilms •Highest amount of *S. aureus* cells after 48 h	24 h: 83.4/16.6 48 h: 54.5/45.5 72 h: 12/88
*P. aeruginosa* PAO1 + *C. albicans* SC5314	•PA01 on bottom and *C. albicans* on top •Highest amount of PAO1 cells after 48 h •*C. albicans* cells decrease over time	24 h: 55/45 48 h: 84/16 72 h: 84.6/15.4
*S. aureus* SH1000 + *P. aeruginosa* PAO1	•*S. aureus* almost completely lysed •PA01 forms a strong multicellular layer on the bottom	24 h: 23.4/76.6 48 h: 1.4/98.6 72 h: 0/100
Triple species		
*S. maltophilia* K279a/sm454 + *S. aureus* SH1000 + *C. albicans* SC5314	•K279a builds the first bottom layer, followed by *S. aureus* and *C. albicans* •Only few cells of *S. aureus* are present in the biofilm •After 48 h, *C. albicans* builds hyphae, which are growing towards the top of the biofilm	24 h: 51/23.8/25.2 48 h: 44.3/25/30.7 72 h: 44.5/42.6/12.9
*S. maltophilia* K279a/sm454 + *P. aeruginosa* PAO1 + *C. albicans* SC5314	•K279a builds the first bottom layer •Cell amount of K279a increases over time •*C. albicans* builds hyphae, which are growing towards the top of the biofilm	24 h: 28.5/42.9/28.6 48 h: 28/32/40 72 h: 24.2/45.5/30.3
*S. maltophilia* K279a/sm454 + *S. aureus* SH1000 + *P. aeruginosa* PAO1	•K279a builds the first bottom layer •Cell amount of K279a increases over time •Cell amount of *S. aureus* decreases over time •*S. aureus* forms a thin layer directly after the first layer of K279a •No complete lysis of *S. aureus*, although *P. aeruginosa* is nearby •Cell numbers of *P. aeruginosa* increase over time	24 h: 32.4/2.9/64.7 48 h: 40.5/2,7/56.8 72 h: 65.2/4.4/30.4

#### Layer and niche formation are a key to dual and triple-species biofilms

Both *S. maltophilia* strains SM454 and K279a in combination with PAO1 always formed distinct layers of populations within the biofilm. This applied to 24-, 48- and 72-h-old biofilms. SM454 and K279a formed always the first layer at the bottom of the biofilm, and PAO1 formed the upper layer of the biofilm. This was observed for biofilms grown under flow conditions and under static conditions but was most pronounced in flow-cell-grown films (Fig. 2G). Notably, *P. aeruginosa* cells always dominated the biofilms by cell numbers, and this did not change over time (Fig. 2; Table 2). Further CLSM analysis of dual-species biofilms of *S. maltophilia* strain K279a and *S. aureus* SH1000 implied a distinct three-dimensional structure with a three-layered sandwich-like model (Fig. 2; Table 2). *S. maltophilia* K279a forming the bottom layer; *S. aureus*, the mid layer; and *S. maltophilia* K279a, the top layer. The reason behind this particular species spatial distribution remains unclear but indicates that the different species tend not to mix well in these biofilms but rather formed niches. This might be a strain-specific trait, since this sandwich-like structure was mainly observed for K279a and less prominent in films grown with SM454.

Further image analysis of dual-species biofilms of *S. maltophilia* K279a and the fungi *C. albicans* showed strong attachment of K279a cells to the *C. albicans* hyphae. This resulted in a clustering of K279a cells around the hyphae of *C. albicans* over time (Fig. 2; Table 2). Surprisingly, SM454 did not attach to the hyphae, indicating that this might yet be another strain-specific trait.

We further established three different combinations of triple-species biofilms: (i) *S. maltophilia*, *S. aureus*, and *P. aeruginosa*; (ii) *S. maltophilia*, *S. aureus*, and *C. albicans*; and (iii) *S. maltophilia*, *P. aeruginosa*, and *C. albicans.*

**Fig 3:**
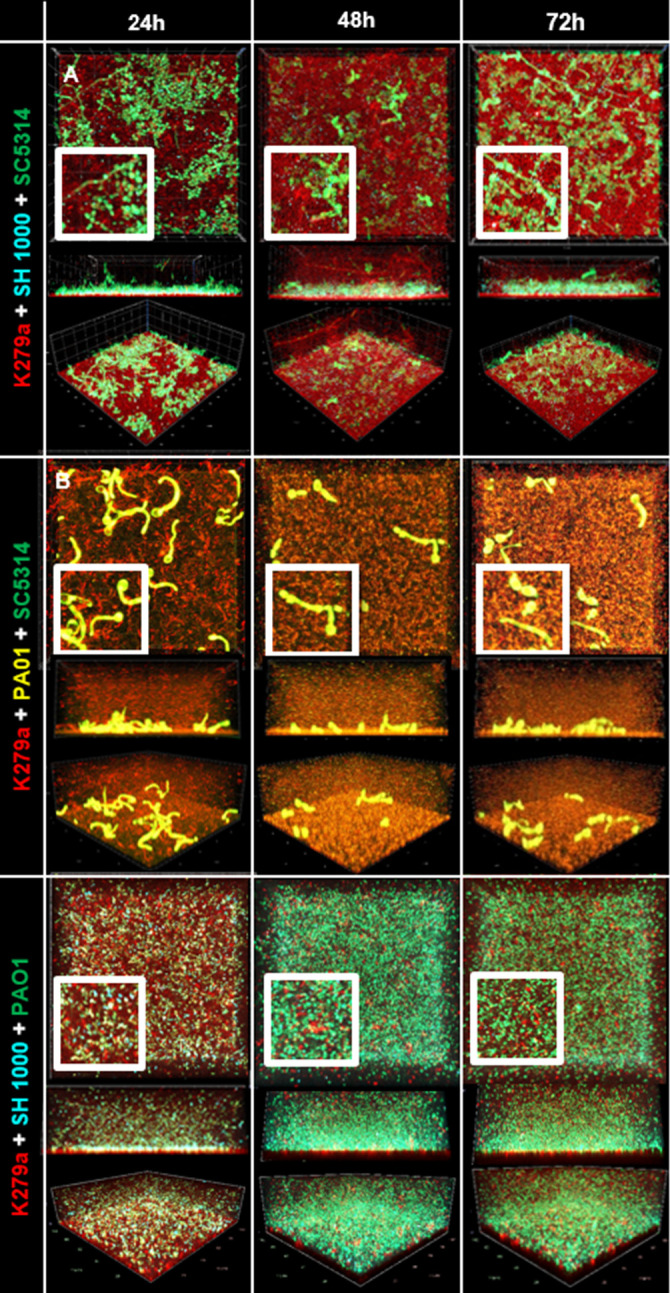
Confocal microscopy images of triple-species biofilms were grown under static conditions at 37°C in 10% LB. Images were taken after 24 h, 48 h, and 72 h. (**A**) Triple-species biofilm of *S. maltophilia* K279a tdTomato (red) + *S. aureus* SH1000 AmCyan (cyan) *+ C. albicans* SC5314 sfGFP (green). (**B**) Triple-species biofilm of *S. maltophilia* K279a tdTomato (red) + *P. aeruginosa* PAO1 eYFP (yellow) + *C. albicans* SC5314 sfGFP (green). (**C**) Triple-species biofilm of *S. maltophilia* K279a tdTomato (red) + *S. aureus* SH1000 AmCyan (cyan) + *P. aeruginosa* PAO1 GFP (green).

Notably, in all triple-species biofilms, *S. maltophilia* always formed the first layer at the bottom (Fig. 3). In the triple-species biofilms with *P. aeruginosa* present, *P. aeruginosa* appeared to overgrow the other species. The filamentation of *C. albicans* was also strongly reduced in the presence of *P. aeruginosa* over time. Similar effects have been reported for co-culture experiments of *P. aeruginosa* and the black yeast *Exophiala dermatitidis* in cystic fibrosis (CF)-like environment, and it was shown that the quorum-sensing molecules N-acyl homoserine lactone (AHLs) of *P. aeruginosa* play a major role in this phenotype (55).

Although the number of cells of *S. aureus* was strongly reduced in this triple-species biofilm, very few *S. aureus* cells were still detectable (Table 2; Fig. 3).

Image analysis of the triple-species biofilm *S. maltophilia* K279a, *S. aureus* SH1000 and *C. albicans* SC5314 revealed again the attachment of K279a cells to the hyphae of *C. albicans*.

**Fig 4:**
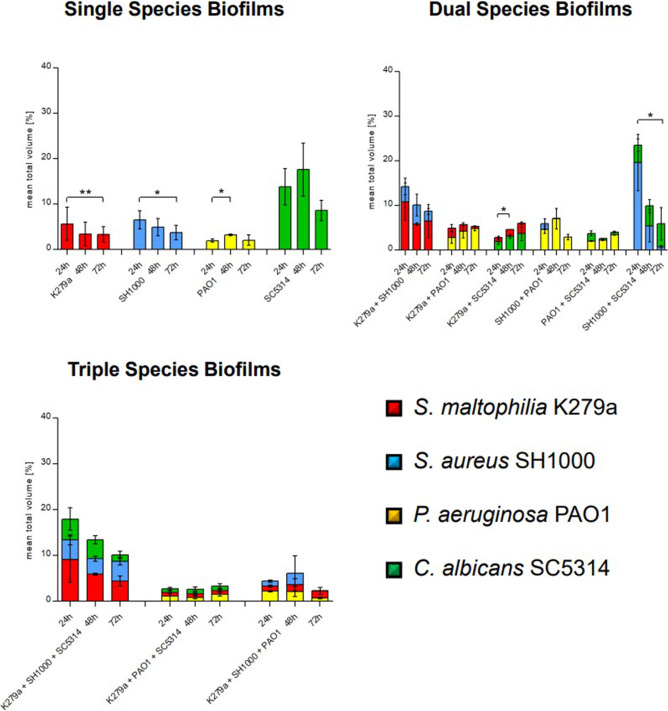
Mean total volume of single-, dual-, and triple-species biofilms indicating species and time-dependent growth behavior. Biofilms were cultivated in LB 10% with a start OD of 0.05 in Ibidi chamber slides under static conditions. After 24 h, 48 h, and 72 h at 37°C, the mean pixel volume of three samples per species was calculated as described in Materials and Methods. *P-value <0.05; **P-value <0.001.

Our data further imply that the volume of bacterial cells was significantly affected when the different species grew in dual biofilms as compared to when they grew alone. We observed that *P. aeruginosa* dominated dual-species biofilms based on the volume occupied and when we used the Fiji software to analyze the cell volume/ratios. These concerned biofilms formed with K279a, *C. albicans*, and *S. aureus.* The total volume occupied by *S. maltophilia* cells was significantly reduced (>80% reduction) in the presence of *P. aeruginosa* after 24 h (*P* = 0.0042). However, in the presence of *S. maltophilia*, the volume occupied by *P. aeruginosa* cells increased sixfold after 72 h (*P* = 0.0266). The overall volume occupied by *C. albicans* was significantly reduced after 24 h in the presence of *P. aeruginosa* (*P* = 0.0053) and in the presence of *S. maltophilia* K279a (*P* = 0.0059). Image quantification also showed that *S. aureus* can no longer be detected in dual-species biofilms with *P. aeruginosa* after 48 h (Fig. 4; Table 2).

In summary, these data imply that all tested species interact with each other and that niche or layer formation is of importance and is determined on a species and strain level.

#### RNAseq indicates a small set of differentially expressed genes in dual- and triple- versus single-species biofilms

Based on the above-observed cellular interaction of the different pathogens, we were interested in deep RNAseq analyses to identify if and to which extent the presence of the other species would affect expression profiles.

Thereby, a total of five multispecies biofilms were analyzed, of which three were combinations of two strains (dual species) and further two combinations of three strains (triple species) including the yeast *C. albicans* (Table 3). For each sample, three different biological replicates were analyzed with an average of over 10 million mapped reads per replicate generated. As controls, single-species biofilms were analyzed, which were grown under the same conditions.

**TABLE 3:** RNAseq analysis of 72-h dual- and triple-species biofilms of major lung pathogens highlighting certain genes of interest^*a*^

	Differentially expressed genes (%/number)	Up- and downregulated genes (%/number)		Significantly expressed genes with the highest positive log2-fold change and the pathways they are involved in	Significantly expressed genes with the highest negative log2-fold change and the pathways they are involved in
Dual-species biofilms					
*S. maltophilia* K279a +	0.55/25	0.44/20	0.11/5	Lactate utilization; LldR operon BetI, osmoprotection regulation YChF, redox regulated ATPase	Malate and isocitrate lyase (aceA, B) glyoxylate pathway FMN reductase
*S. aureus* SH1000	7.7/229	5.2/137	2.5/92	Serine threonine transporter MarR regulator involved in antibiotic resistance phhospholipase, carboxylesterase starvation inducible binding protein SarV regulator Glyoxylate detoxification	LytR regulator Tryptophane biosynthesis, trpC, trpD, trpA, trpB MarR regulator involved in virulence
					
*S. maltophilia* K279a +	3.35/151	2.66/120	0.69/31	RND transporter Cell wall elongation, RodA Pirin oxidative stress sensor Tranferrin binding protein TonB, TolC proteins Cytochrome oxidase CyoA	FMN reductase Malate and isocitrate lyase (aceA, B) CsBD family proteins Phasin family protein BON family protein Tryptophane biosynthesis
*P. aeruginosa* PAO1	11.6/669	6/356	5.6/313	PA1019a, phenyl acetic acid degradation Various dioxygenases Carnithine degradation pathway MFS transporter for propionic acid tolerance, ABC transporter T3SS secretion negative regulator, PopN	Mg2+ transporter, T4 fimbri, outer membrane protein OprI OprF, LasI, N-acyl-homoserine synthase, VqsM regulator, elastase, LasB, Alginate sigma factor and anti-sigma factor AlgU, MucA, protease LasA, regulator LasR, Cytochrome C1
					
*S. maltophilia* K279a +	0.04/2	0.04/2	0/0	DcaP family trimeric outer membrane transporter, propionate–CoA ligase, prpE	–/–
*C. albicans* SC5314	–/–	–/–	–/–	–/–	–/–
					
Triple-species biofilms					
*S. maltophilia* K279a *+*	0.13/6	0.08/4	0.04/2	Lactate utilization	Malate and isocitrate lyase (aceA, B), glyoxylate pathway
*S. aureus* SH1000 +	5.32/140	3.27/86	2.05/54	Serine/threonine exchange transporter, LAT family SarV → virulence and autolysis	Tryptophan biosynthesis transcriptional regulator, LytTR family transcriptional regulator, MarR family alpha-hemolysin precursor
*C. albicans* SC5314	–/–		–/–	–/–	–/–
					
*S. maltophilia* K279a +	1.54/70	0.95/43	0.59/27	Transporter DcaP family (SMLT_RS211910) Flagellar motor assembly Acetate CoA ligase, propionate metabolism, prpE, pprB, acnD heme exporter protein, CcmD	Spermidine transporter GspM, type II secretion Dicarboxylate transporter BON-domain family protein TetR-regulator CsbD family protein
*P. aeruginosa* PAO1 +	9.37/532	4.07/231	5.3/301	PA3501, hypothetical cdhA, hydoxybutryl-CoA-dehydrogenase antB, antC, anthranilate dioxygenase heme 1 biosynthesis, NirL XyL, dehydrogenase, benzoate degradation	PA 3765, hypothetical Elastase, LasB, Mg2+ transporter, hypothetical PA1041, aphC, alkyl hydroxyperoxide reductase OprI, outer membrane protein, VqsM regulator Fimbrial biosynthesis, CtyC oxidase, PA1983
*C. albicans* SC5314	–/–	–/–	–/–	–/–	–/–

^*a*^ For a detailed list of all differentially regulated genes with log2-fold values, please see Table S5.

**Fig 5:**
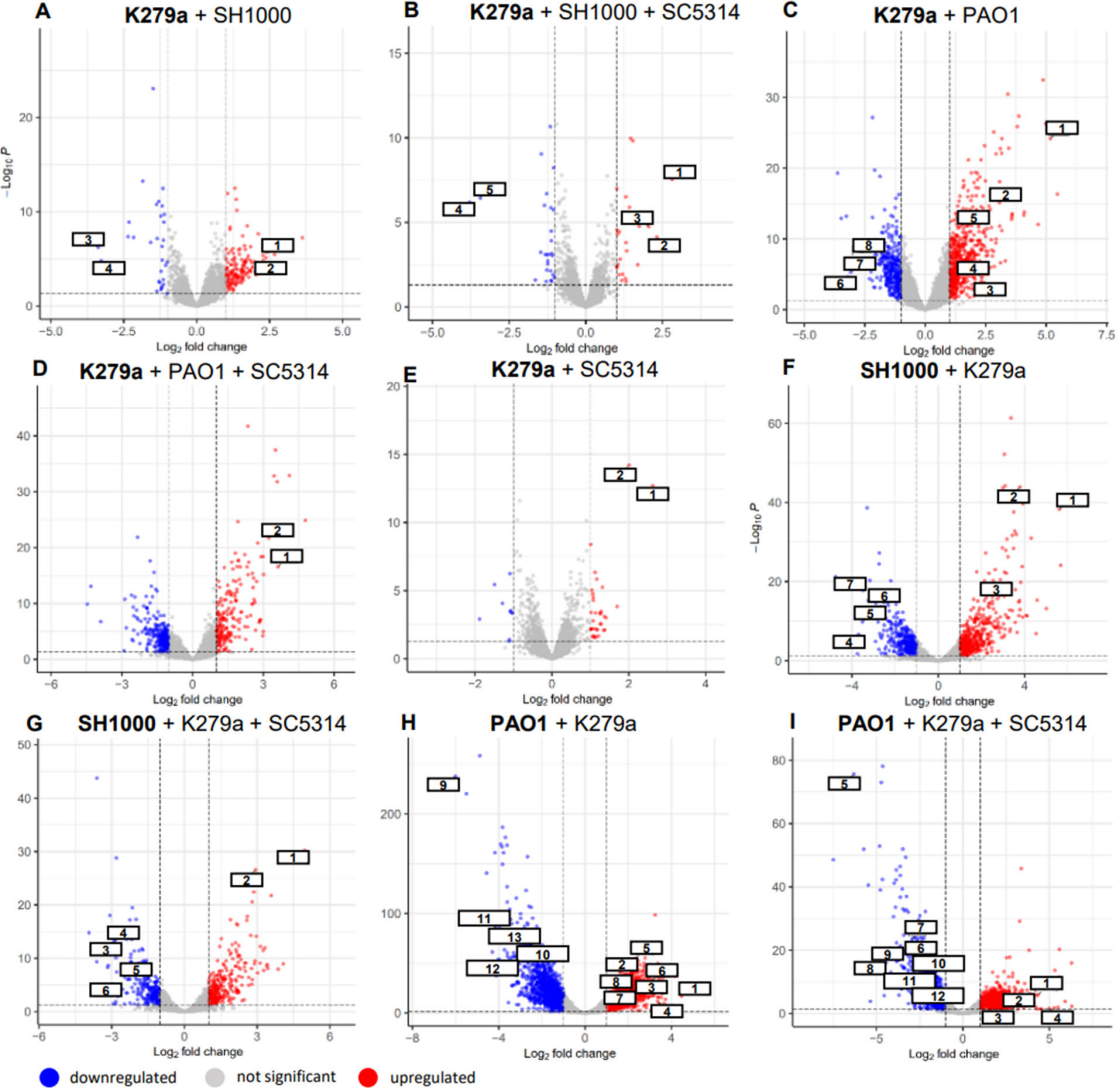
Volcano plots representing the differential gene expression results of RNAseq data. Upregulated genes (log2 fold change ≥2) are represented by red dots, whereas downregulated genes (log2-fold change ≤−2) are represented by blue dots. (**A**) K279a + SH1000: regulated genes of K279a belong to the lactate metabolism [SMLT_RS13840 (**A1**), SMLT_RS13835 (**A2**)] and the glyoxylate cycle [SMLT_RS01085 (**A3**), SMLT_RS01090 (**A4**)]. (**B**) K279a + SH1000 + SC5314: regulated genes of K279a belong to the lactate metabolism [SMLT_RS13840 (**B1**), SMLT_RS13835 (**B2**), SMLT_RS13830 (**B3**)] and the glyoxylate cycle [SMLT_RS01090 (**B4**), SMLT_RS01085 (**B5**)]. (**C**) K279a + PAO1: regulated genes of K279a belong to the shape [SMLT_RS19285 (**C1**)], propionate degradation [SMLT_RS17185 (**C2**)], secretion systems [SMLT_RS13065 (**C3**), SMLT_RS06235 (**C4**)], respiration [SMLT_RS20930 (**C5**)], the glyoxylate cycle [SMLT_RS01090 (**C6**), SMLT_RS01085 (**C7**)], and the tryptophane biosynthesis [SMLT_RS16260 (**C8**)]. (**D**) K279a + PAO1 + SC5314: regulated genes of K279a are a DcaP family trimeric outer membrane transporter [SMLT_RS21910 (**D1**)], a propionate–CoA ligase [SMLT_RS04515 (**D2**)]. (**E**) K279a + SC5314: regulated genes of K279a are a DcaP family trimeric outer membrane transporter [SMLT_RS21910 (**E1**)] and a propionate–CoA ligase [SMLT_RS04515 (**E2**)]. (**F**) SH1000 + K279a: regulated genes of SH1000 are a serine/threonine exchange transporter [SAOUHSC_01450 (**F1**)], the transcriptional regulator sarV [SAOUHSC_02532 (**F2**)], a toxin called YoeB [SAOUHSC_02691 (**F3**)] and the tryptophane biosynthesis [SAOUHSC_01369 (**F4**), SAOUHSC_01368 (**F5**), SAOUHSC_01371 (**F6**), SAOUHSC_01372 (**F7**)]. (**G**) SH1000 + K279a + SC5314: regulated genes of SH1000 are a serine/threonine exchange transporter [SAOUHSC_01450 (**G1**)], the transcriptional regulator sarV [SAOUHSC_02532 (**G2**)], and the tryptophane biosynthesis [SAOUHSC_01371 (**G3**), SAOUHSC_01372 (**G4**), SAOUHSC_01368 (**G5**), SAOUHSC_01369 (**G6**)]. (**H**) PAO1 + K279a: regulated genes of PAO1 are a thioestherase [PA1019a (**H1**)], type 2 [PA2676 (**H2**)], type 3 [PA1698 (**H3**), PA1724 (**H4**)], and type 6 [PA5090 (**H5**)] secretion systems, alginate biosynthesis [PA3549 (**H6**), PA3546 (**H7**)], exotoxin A [PA1148 (**H8**)], virulence factors [PA3724 (**H9**), PA1871 (**H10**)], and quorum-sensing-related genes [PA1432 (**H11**), PA2227 (**H12**), PA3476 (**H13**)]. (**I**) PAO1 + K279a + SC5314: regulated genes of PAO1 are a thioestherase [PA1019a (**I1**)], type 2 [PA0684 (**I2**)], and type 3 [PA1718 (**I3**)] secretion systems, type III export protein PscG (**I4**), virulence factors [PA3724 (**I5**), PA1871 (**I6**)], transcriptional regulator RhlR (**I7**) quorum-sensing-related genes [PA2227 (**I8**), PA1432 (**I9**)], and genes involved in aerobic respiration [PA0105 (**I10**), PA0108 (**I11**), PA0106 (**I12**)].

Table S1 and S2 give an overview on the obtained data and differentially expressed genes (log2-fold change ≥2 or ≤−2), and Table 3; Table S5; Fig. 5 summarize the main findings and most strongly differentially regulated genes.

Perhaps, one of the most intriguing findings was that we detected major differences in the gene expression profiles of each bacterial species in a mixed biofilm as compared to single-species biofilm (Table 3). The total number of differentially expressed genes for *S. maltophilia* with 151 genes (3.3%) was highest when *P. aeruginosa* was present and lowest with two genes (0.04%) when only *C. albicans* was present. In general, *P. aeruginosa* displayed the strongest response in our mixed biofilm model with 669 (11.8%) differentially regulated genes in the presence of *S. maltophilia.* A total of 138–229 (5%–7%) of *S. aureus* genes were differentially regulated in the presence of *S. maltophilia* and *C. albicans* (Table 3).

The RNAseq data implied that each bacterial species reacted to the presence of the other species and that each species had significant impact on the metabolism of the other species.

**Fig 6:**
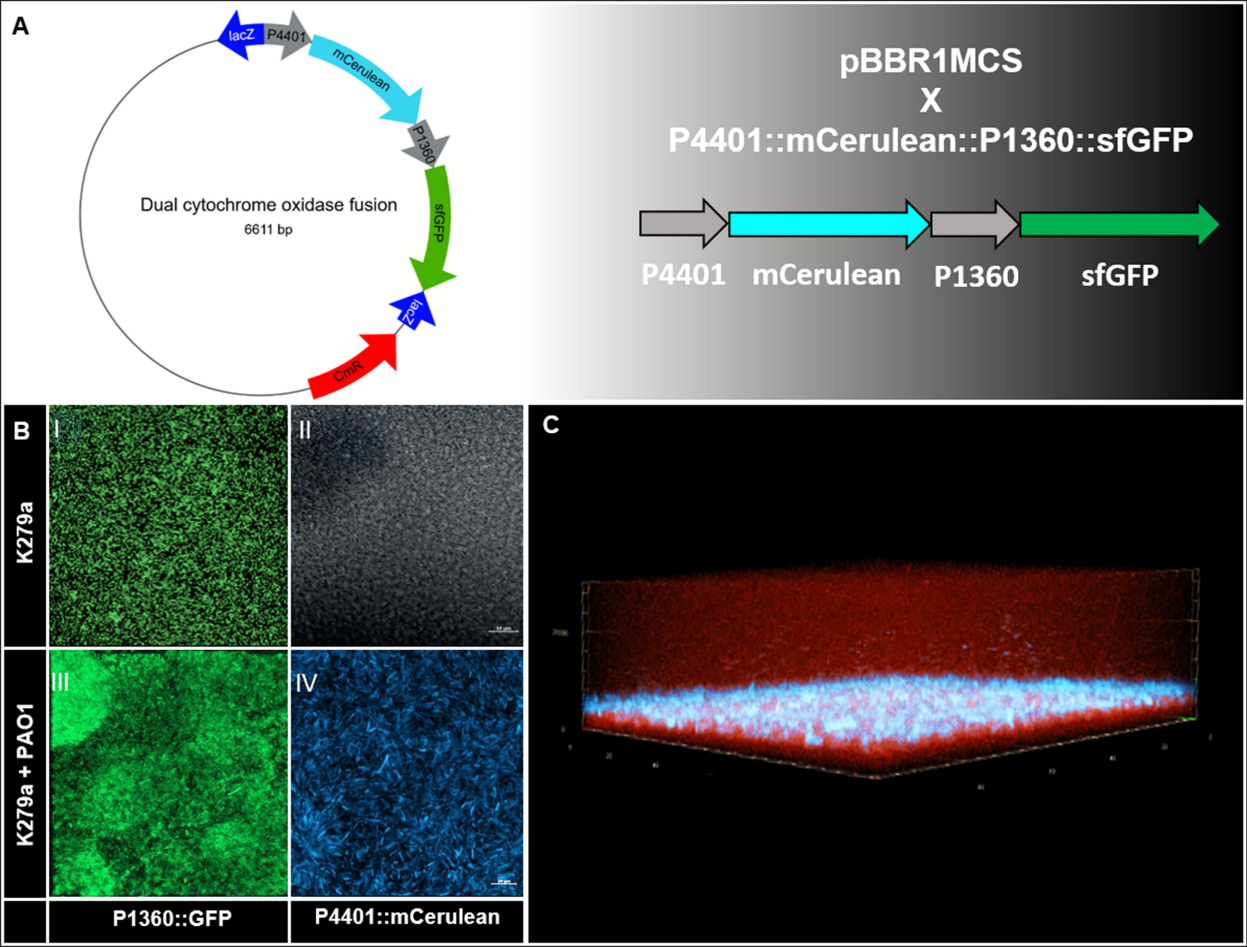
Promotor fusion construct pBBR1MCS::P4401::mCerulean::P1360::GFP reveals different respiration systems in *S. maltophilia* K279a in single versus co-culture. (**A**) Genetic map of the promotor fusion construct pBBR1MCS::P4401::mCerulean::P1360::GFP. When growing alone, only Cyt 1360 is strongly expressed in K279a (BI, BII), whereas in co-biofilm with PAO1, the Cyt 4401 is additionally also highly expressed (BIII, BIV). (**C**) Dual-species biofilm of K279a × pBBR1MCS::P4401::mCerulean and PAO1 mCherry. Cyt 4401 (blue) is highly expressed, and the mCerulean signal correlates with the layer of K279a in the middle of the biofilm.

We noticed that in general, competition for nutrients (Mg^2+^, Fe^2+^, and/or Fe^3+^, phosphorous, and small molecules) was of importance for the bacteria living in dual- and triple-species biofilms, and therefore, transporters were often differentially regulated (Table 3; see Fig. 6).

Furthermore, the majority of genes differentially transcribed in the multispecies biofilms were involved in respiration, secretion, propionate, and lactate metabolism.

Thereby, in mixed biofilms of K279a and *S. aureus*, it was evident that genes involved in lactate metabolism such as the lactate permease (SMLT_RS13840), FMN-dependent L-lactate dehydrogenase LldD (SMLT_RS13830), and the transcriptional regulator LldR (SMLT_RS13835) were significantly and most strongly upregulated in K279a (Table 3; Table S2). On the other hand, serine/threonine exchange transporter, LAT family

(SAOUHSC_01450) of *S. aureus* was upregulated in dual biofilms with *S. maltophilia* as well as a phospholipase (SAOUHSC_02824), hypothetical proteins, and SarV (a regulator involved in the autolysis of *S. aureus* (SAOUHSC_02532).

Within this framework, it had previously been shown that *S. aureus* can excrete large amounts of lactate to maintain the redox balance (56), and co-culture experiments of *P. aeruginosa* and *S. aureus* showed that *P. aeruginosa* drives *S. aureus* to a fermentative lifestyle which leads to the production of lactate that is then metabolized by *P. aeruginosa* (57). Additional lactate measurements in biofilms supernatants confirmed these findings in part and suggested that lactate appears to be a major metabolite in *S. maltophilia*, *S. aureus*, and *C. albicans* single- and multispecies-biofilm supernatants. Lactate concentrations were in the range of 1.4–130.5 µM (Fig. S2).

Similarly, in multispecies *S. maltophilia* biofilms malate synthase (SMLT_RS01085) and isocitrate lyase (SMLT_RS01090), both key genes of the gyloxylate pathway were strongly downregulated (Table 3; Table S2; Fig. 5). Since the glyoxylate pathway is used to incorporate C2 molecules into the cell metabolism, the downregulating implies that enough other carbon sources were available for anabolism in K279a.

Notably, Gram-negative bacteria often harbor various cytochrome oxidases that allow adaption to varying oxygen concentrations (58 – 60). K279a codes in its genome for at least three cytochrome oxidases (*cyt1-3*) (54), and PAO1, for five cytochrome oxidases (*cbb_3_-1*, cbb_3_-2, aa_3_, Cyo, and CIO) (61, 62)

We observed that K279 switched between the different cytochrome oxidases depending on single-species/multispecies biofilm or planktonic lifestyle. K279 mainly employs the cytochrome oxidase Cyt1 (smlt3282-3287) under planktonic conditions in aerobic environments and the Cyt2 (smlt1360-1363) under single-species biofilm lifestyle (19). In dual-species biofilms, however, it strongly transcribes the Cyt3 (*cyoA-C*, smlt 4397–4402) as the main cytochrome oxidase. The latter is a quinol oxidase (Table 3). This switching was even more enhanced in flow-cell-grown biofilms and resulted in an up to 70-fold increased transcription (data not shown).

Intrigued by the observation that K279a switched between different cytochromes, we asked if it would be possible to monitor the expression of the cyoA (*cyoA-C*, smlt 4397–4405) gene in dual-species biofilms versus single-species biofilms and within the typical layer that was formed by K279a. For this, we constructed a reporter fusion harboring the mCerulean gene fused with the promoter of the K279a *cyoA* (SMLT_4401, operon 4399–4405) and the promoter of the cytochrome CytC2 (SMLT_1360) in the medium copy vector pBBRMCS-1 (Fig. 6).

Using this construct, we were able to confirm the strong expression of *cyoA* in dual-species biofilms within the distinct layers of K279 cells. Interestingly, the CytC2 (SMLT_1360) fusion was only expressed in single- and dual-species biofilms but not in liquid culture (Fig. 6).

One of the most striking observations was that the co-cultivation of K279 and PAO1 resulted in a partial attenuation of several of the AI-I and N-acyl-homoserine lactone-dependent quorum sensing (QS) regulatory circuits, LasI/R in PAO1. The LasI/R regulon in PAO1 is one of two N-Acyl-homoserine-lactone-dependent regulatory circuits in part controlling pathogen-related processes (63, 64). LasI and LasR were strongly downregulated. In addition, the main QS regulatory protein VqsM and the pathogen-related elastase LasB and proteases LasA were strongly affected in their transcription levels (Table 3) in dual-species biofilms compared to single-species biofilms.

This observation suggests that K279a is able to interfere with cell-cell communication mechanisms on a community-wide level in PAO1. So far, not much is known about quorum-quenching activity by *S. maltophilia*. Some strains have been reported to have AHL degrading enzymes (65). Another study has demonstrated that the signaling molecule DSF Cis-9-octadecenoic acid can show quorum-quenching and antibiofilm activity (66). These are possible mechanisms that could be at play in this case of our observation. However, this requires further investigation.

Altogether, these data imply a significant influence of *S. maltophilia* on the gene expression of *S. aureus*, *P. aeruginosa*, and *C. albicans*, when grown together in mixed-species biofilms and possible interspecies interaction in mixed biofilms. Our data suggest that some of these key interactions in our mixed-species biofilm model are involved in lactate metabolism, respiration, and quorum sensing.

We observed that genes involved in lactate metabolism were differently upregulated in K279a. We also detected lactate in the supernatant of these biofilms (Fig S2).

Another interesting finding was the upregulation of one of the cytochrome oxidase clusters (cyoA - C, smlt 4397–4402) in *S. maltophilia* K279a in the presence of *Pseudomonas aeruginosa*. This suggest that *P. aeruginosa* interferes with the respiration of *S. maltophilia* K279a. The molecular mechanism behind this, however, still needs to be elucidated.

A further striking observation was that *S. maltophilia* probably interferes with the signaling system of *P. aeruginosa* which led to a downregulation of QS genes such as the autoinducer synthase lasI (PA1432) and rhlL (PA3476). The transcription of QS-dependent pathogen-related elastase LasB und LasA of *P. aeruginosa* was, therefore, also strongly affected. Nonetheless, further experiments are required to understand the mechanism behind this.

**Fig 7:**
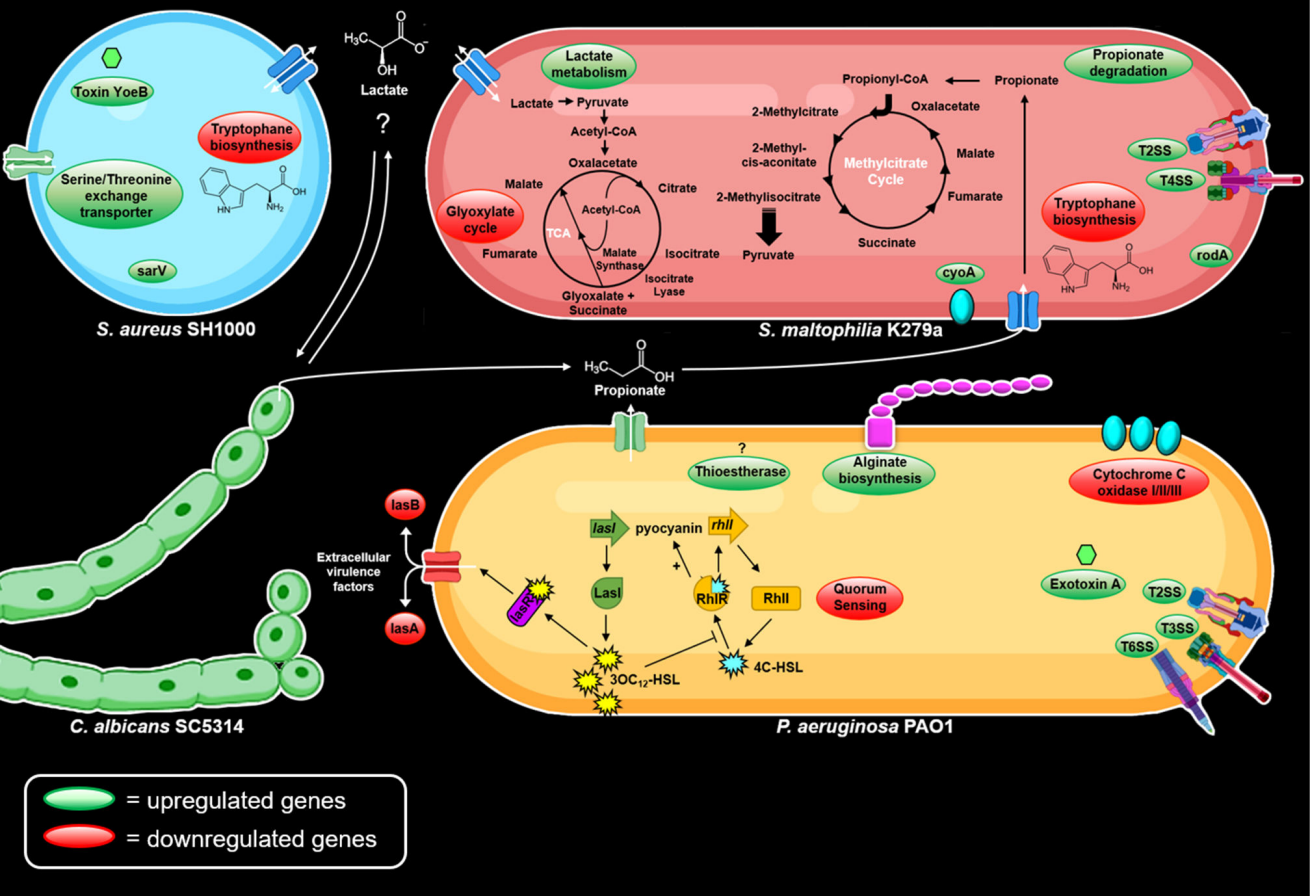
Possible interactions between the different species used in this study. RNAseq analysis of biofilms, which were cultivated in 10% LB in Ibidi slide chambers at 37°C for 72 h, revealed a differentially gene expression of each species in dual- and triple-species biofilms as compared to single-species biofilms.

In Fig. 7, we demonstrate the possible interactions among the different species in our mixed-species biofilm models.

## DISCUSSION

Only relatively few studies have been published analyzing the interactions of major pathogens in laboratory-grown multispecies biofilms (13, 67, 68). These studies have mainly focused on the interaction between two species (69, 70) and only few with three (71 – 73) and four species (6, 74). Most studies focused on *P. aeruginosa* combined with *S. aureus* (75, 76) and/or *C. albicans* (77, 78). *S. maltophilia* is a pathogen that is clinically relevant today. However, only very few studies have combined this emerging pathogen with others (73, 79, 80). One interesting study, however, has shown that when co-infected with *P. aeruginosa*, *S. maltophilia* counts increase significantly in lung samples and bronchoalveolar lavages of mice, with a direct correlation to the density of *P. aeruginosa* population. The study also revealed that *S. maltophilia* and *P. aeruginosa* form layered biofilms *in vitro* and colocalize in the lungs during dual-species infection. Furthermore, it was pointed out that active cellular processes by *P. aeruginosa* provide a substantial advantage to *S. maltophilia* during polymicrobial infections (81).

Even though, today, *S. maltophilia* is frequently co-isolated with other pathogens such *P. aeruginosa* and *S. aureus* from infection sites like wounds or lungs, very little is still known about the impact of *S. maltophilia* on the gene expression of these other pathogens and vice versa.

Within these settings, we constructed and employed a multitude of fluorescently tagged variants of these pathogens allowing co-cultivation of them in different color combinations in a ratio of 1:1, and we quantified the individual species within various multispecies biofilms (Fig. 1 and 2). The image analyses of mixed-species biofilms provided strong evidence that the four different species used in this study competed for space and room. This lead in part to niche formation which was observed as a distinct layer formation. *S. maltophilia* K279a produced sandwich-like layers in dual-species biofilms with *S. aureus*; it was the first to colonize the surface, and it formed a top layer (Fig. 2). These sandwich-like structures have not been previously described in multispecies biofilms. Furthermore, it was noteworthy that *S. aureus* was the least competitive microorganism (Fig. 2 and 3) and usually overgrown by all other microorganisms. It has previously been shown that layer formation can occur in multispecies biofilms and spatial organization is a reflection of interspecies interactions; however, the molecular mechanisms behind this are not yet fully understood (82, 83).

The transcriptome data implied that in the dual- and triple-species biofilms, a rather small set of specific genes was differentially transcribed compared to when the species grow alone in single-species control biofilms.

In general, genes involved in Mg^2+^, Fe, and phosphorous uptake were of importance for the bacteria living in dual- and triple-species biofilms, and because of this, transporters were often differentially regulated. The data further imply that the low oxygen availability is a major driver and either induces a fermentative metabolism and/or that under the mixed-species conditions, different cytochrome C oxidase is activated compared to life in aerobic cultures and single-species biofilms. In *S. maltophilia*, a third cytochrome C oxidase was activated only under the dual-species biofilm conditions, and we were able to verify this by using fluorescently labeled variants of the different cytochrome C oxidases (Fig. 7). This was concurrent with the observation of the layer and sandwich-like structures.

Fig. 6 summarizes and highlights major genes and pathways identified in this study that are involved in the possible interaction of the four major pathogens.

Interestingly, in dual-species biofilms consisting of K279 and PAO1, the QS and N-Acyl-homoserine lactone-dependent circuit was attenuated in PAO1. This resulted in strong downregulation of LasB and LasA and other genes usually controlled by the LasI/LasR regulon (Table 3). This observation may indicate a specific quorum-quenching mechanism employed by K279a on the LasI signaling molecule (C12-N-Acyl homoserine lactone) (84).

While we have identified multiple key features and differentially regulated genes and pathways (Fig. 6; Table 3) in these multispecies consortia, a potential drawback arises from the fact that the data were generated using a laboratory flow and/or static biofilm system. The environmental conditions in our models certainly differ significantly from those in lungs or wounds but allow a first glimpse into life in mixed-species biofilms. However, further experiments are required under conditions mimicking *in vivo* environments. For instance, growing the biofilms in artificial sputum media which mimics the CF lung environment as well under low oxygen levels.

Nevertheless, the data here provide first evidence that the gene expression profiles of *S. maltophilia*, *P. aeruginosa*, and *S. aureus* is affected in our multispecies biofilm model under these *in vitro* conditions. We also observed that species- and strain-specific traits are of importance in these interactions. Future work will now have to elucidate the molecular mechanisms underlying these interactions in more detail.

### Conclusions

This is one of the first studies analyzing in detail the structural, phenotypic, and genotypic traits of multispecies biofilms employing pathogens relevant in lung infections. LSM images revealed strain- and species-specific phenotypic traits such as sandwich-like layer formation in our mixed-species biofilm models and the displacement of some species by others in the biofilms. Additional RNAseq data imply that there is strong competition for nutrients (Fe^2+^, P_i_, Mg^2+^) as well as a shift in metabolic and respiratory pathways toward fermentative modus. These data will ultimately be of value for the identification of novel drug targets for treatment of mixed-species pathogenic biofilms.

## Data Availability

Sequence data reported in this publication have been submitted to NCBI/ENA/DDBJ. They are publicly available under accession PRJEB56214. All plasmids used in the mini Tn7T labelling approach have been deposited in the NCBI database under the accession numbers: OQ253286–OQ253289, OP566392,OP566393, and OP566395.

## SUPPLEMENTAL MATERIAL

Fig S1 spectrum.00859-23-s0001.pdf Single species biofilms grown under static conditions at 37°C in 10% LB and analysed with confocal microscopy.

Fig S2 spectrum.00859-23-s0002.pdf Lactate measurement of different biofilm supernatants reveals a species dependent lactate secretion.

Table S1 spectrum.00859-23-s0003.xlsx Overall transcriptome data.

Table S2 spectrum.00859-23-s0004.xlsx transcriptome data up and downregulated genes.

Table S3 spectrum.00859-23-s0005.xlsx Microscopy settings.

Table S4 spectrum.00859-23-s0006.docx Primers used in this study.

Table S5 spectrum.00859-23-s0007.xlsx Highlighted genes in the volcano plots.

Table S6 spectrum.00859-23-s0008.xlsx Vertis Data delivery note.

Table S7 spectrum.00859-23-s0009.xlsx Quantification of the LSCM images.

ASM does not own the copyrights to Supplemental Material that may be linked to, or accessed through, an article. The authors have granted ASM a non-exclusive, world-wide license to publish the Supplemental Material files. Please contact the corresponding author directly for reuse.
