# Supplementary material for: Stenotrophomonas maltophilia affects the gene expression profiles of the major pathogens Pseudomonas aeruginosa and Staphylococcus aureus in an in vitro multispecies biofilm model
Source: Microbiol Spectr. 2023 Oct 11;11(6):e00859-23. doi: 10.1128/spectrum.00859-23 (PMC10714729; doi:10.1128/spectrum.00859-23)
Supplement: Fig S1 — Single species biofilms grown under static conditions at 37°C in 10% LB and analysed with confocal microscopy. [file spectrum.00859-23-s0001.pdf]

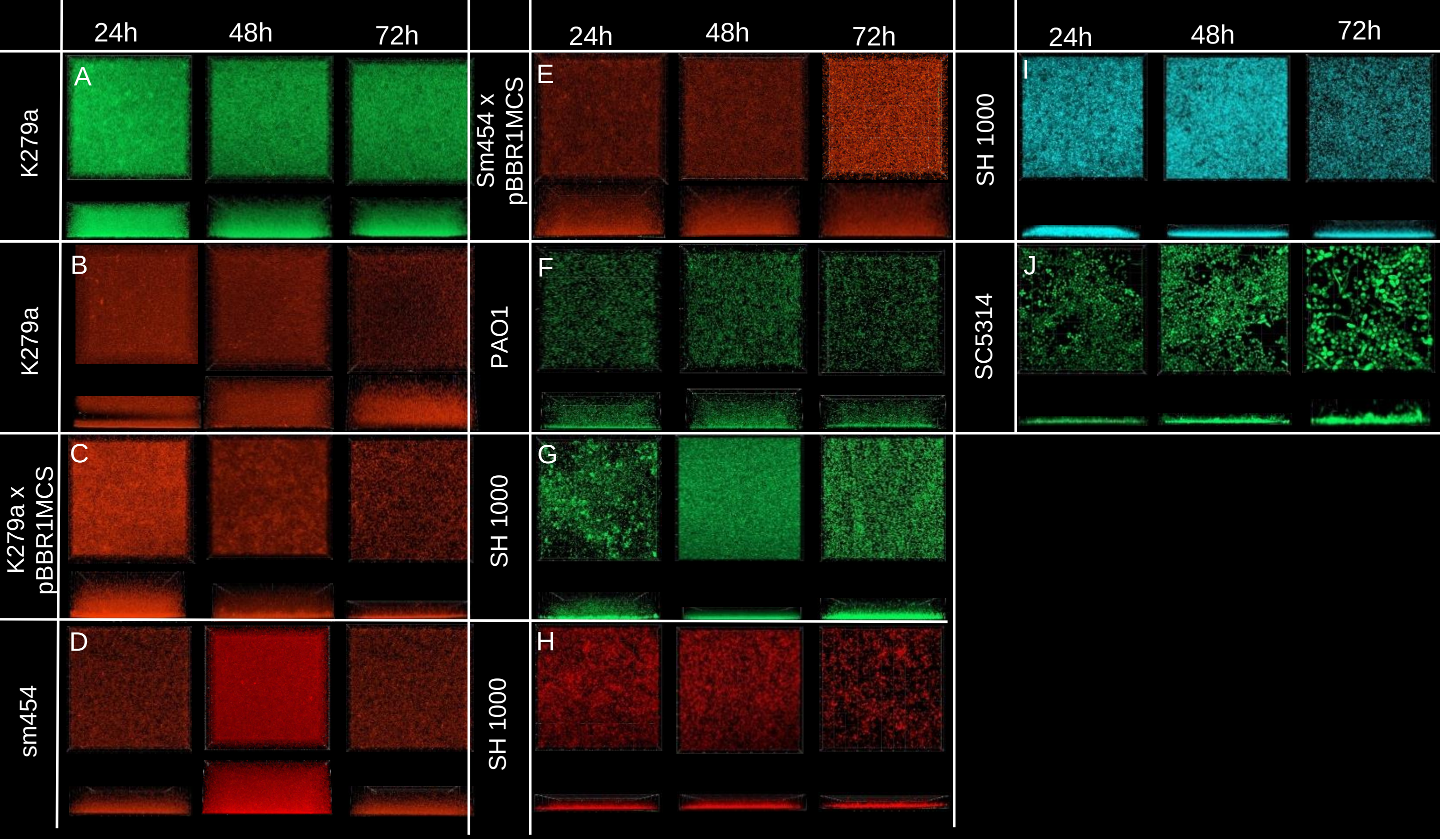

FIGURE S1: Single species biofilms grown under static conditions at 37°C in 10% LB and analysed with confocal microscopy. Images were taken after 24h, 48h and 72h. **(A)** *S. maltophilia* K279a GFP. **(B)** *S. maltophilia* K279a tdTomao. **(C)** *S. maltophilia* K279a x pBBR1MCS tdTomato. **(D)** *S. maltophilia* sm454 tdTomato. **(E)** *S. maltophilia* sm454 x pBBR1MCS tdTomato. **(F)** *P. aeruginosa* GFP. **(G)** *S. aureus* SH1000 sfGFP. **(H)** *S. aureus* SH1000 mCherry. **(I)** *S. aureus* SH1000 AmCyan. **(J)** *C. albicans* SC5314 sfGFP.
