# Supplementary material for: Stenotrophomonas maltophilia affects the gene expression profiles of the major pathogens Pseudomonas aeruginosa and Staphylococcus aureus in an in vitro multispecies biofilm model
Source: Microbiol Spectr. 2023 Oct 11;11(6):e00859-23. doi: 10.1128/spectrum.00859-23 (PMC10714729; doi:10.1128/spectrum.00859-23)
Supplement: Fig S2 — Lactate measurement of different biofilm supernatants reveals a species dependent lactate secretion. [file spectrum.00859-23-s0002.pdf]

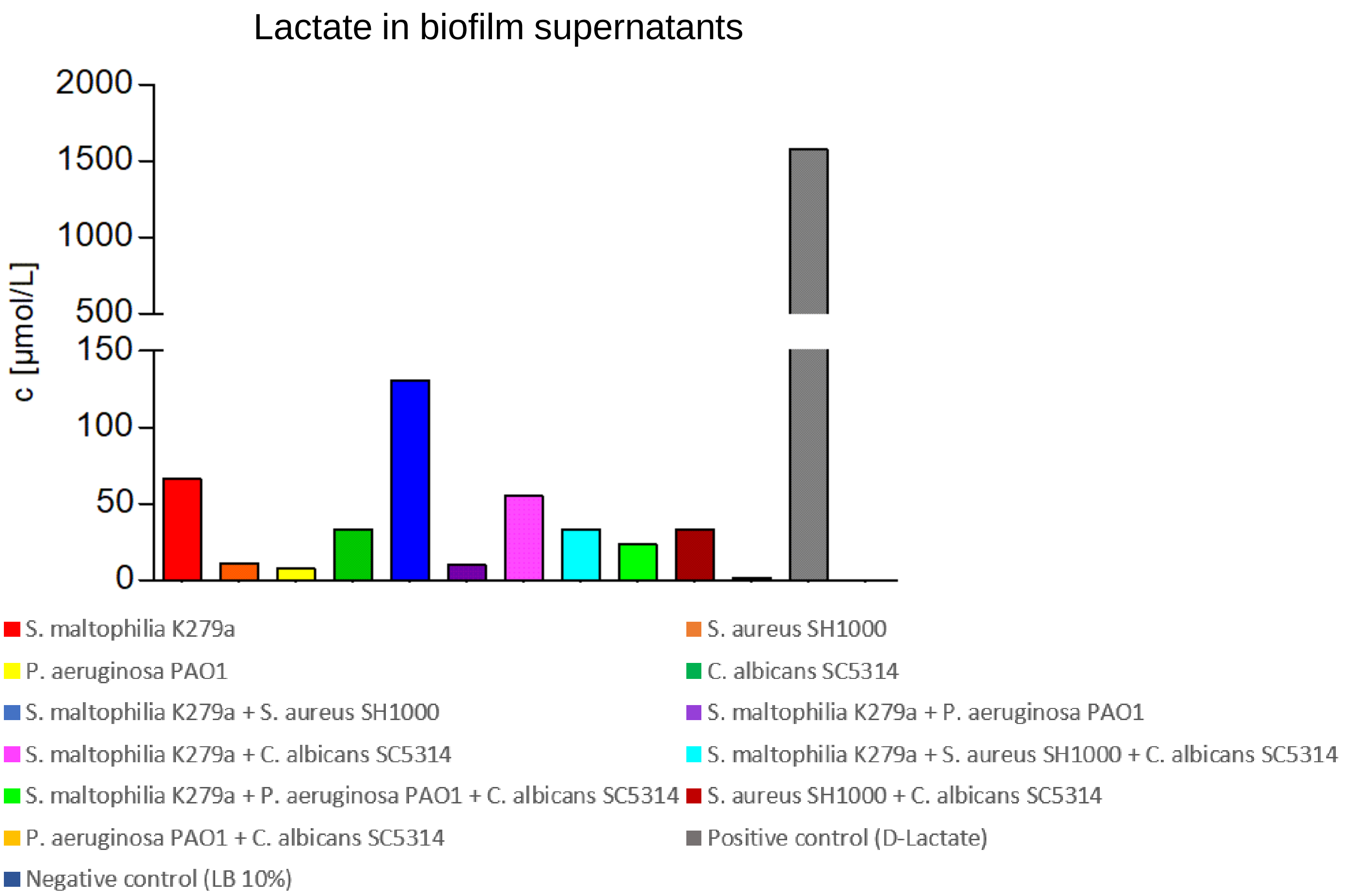

FIGURE S2: **Lactate measurement of different biofilm supernatants reveals a species dependent lactate secretion.** Biofilms were grown under static conditions for 72 hours at 37°C in LB 10%. Supernatants were collected and sterilized via a 0.22 µm filter from clear line. The lactate measurement was performed with the D-Lactic acid/L-Lactic acid kit from BOEHRINGER MANNHEIM / R-BIOPHARM according to the manufacturer‘s protocol.
