# Supplementary material for: Stenotrophomonas maltophilia affects the gene expression profiles of the major pathogens Pseudomonas aeruginosa and Staphylococcus aureus in an in vitro multispecies biofilm model
Source: Microbiol Spectr. 2023 Oct 11;11(6):e00859-23. doi: 10.1128/spectrum.00859-23 (PMC10714729; doi:10.1128/spectrum.00859-23)
Supplement: Table S4 — Primers used in this study. [file spectrum.00859-23-s0006.docx]

| **Primer** | **Sequence [5‘-3‘]** |
| --- | --- |
|  |  |
| smlt for | GCAAGGACAAGGCGATGACCATC |
| smlt rev | CCCCACCACGATTTCATCAGGC |
| *S. aureus* for | AAAGCGATTGATGGTGATACGGTT |
| *S. aureus* rev | CCAAGCCTTGACGAACTAAAGC |
| *P. aeruginosa* for | GCTTCCCTCGCAGAGAAAACATC |
| *P. aeruginosa* rev | GGCCTTGATCCGCCTTCAGG |
| *C. albicans* for | GCTCCTGCTCCTGAAATGAC |
| *C. albicans* rev | CTGGAGCAATTGGTGAGGTT |
| PTn7R | CACAGCATAACTGGACTGATTTC |
| PAO1-glmS-Ctrl1 | GCTGAAGCTCAAGGAAATTTCC |
| PTn7L | ATTAGCTTACGACGCTACACCC |
| PAO1-PA5548-Ctr | TTACCTGCGACGTTATCTGAAGC |
| Smlt4098-Ctrl1 | CATCGTCCTTCATCACCACCA |
| Smtl4099-Ctrl1 | AAATCTCCTACATCCACGCC |
| EYFP_for_BamHI | GGGGATCCATGGTGAGCAAGGGCGAGGA |
| EYFP_rev_SacI | GGGAGCTCTTACTTGTACAGCTCGTCCATGCC |
| mOrange_for_BamHI | GGGGATCCATGGTGAGCAAGGGCGAGGA |
| mOrange_rev_SacI | GGGAGCTCCTACTTGTACAGCTCGTCCATGCC |
| mCerulean_for_BamHI | GGGGATCCATGGTGAGCAAGGGCGAGGAG |
| mCerulean_rev_SacI | GGGAGCTCTTACTTGTACAGCTCGTCCATGC |
| tagBFP_for_BamHI | GGGGATCCATGAGCGAGCTGATTAAGGAGA |
| tagBFP_rev_SacI | GGGAGCTCTTAATTAAGCTTGTGCCCCAGTTTG |
| P1360_for_hindIII | AAGCTTCGGGGCCTTCCTCCTGTGCCGAG |
| P1360_rev_xhoI | CTCGAGCGGTCGATCCAATTGCGAGG |
| sfGFP_for_xhoI | AAGCTTCGGGGCCTTCCTCCTGTGCCGAG |
| sfGFP_rev_kpnI | CTCGAGCGGTCGATCCAATTGCGAGG |
| P4401_for_SacI | GCGAGCTCCGGTCAGTTTTTTCCATACG |
| P4401_rev_xbaI | GCTCTAGATGGAGGTCAACCCTGTTGA |
| mCerulean_for_xbaI | GCTCTAGAATGGTGAGCAAGGGCGAGG |
| mCerulean_rev_BamH | GCGGATCCTTACTTGTACAGCTCGTCCATGCC |
| pCM-sarA_fwd | Taagaattcgtaatcatgtcatag |
| pCM-sarA_rev | Aaataatcatcctcctaagg |
| mCherry_ sarA_fwd | ccttaggaggatgattatttATGGTGAGCAAGGGTGAG |
| mCherry_rev | ctatgacatgattacgaattGAATTCCTACTTGTACAGC |
| AmCyan_sarA_fwd | CCTTAGGAGGATGATTATTTATGGCTCTTTCAAACAAG |
| AmCyan_rev | ctatgacatgattacgaattGAATTCTTAAAAAGGAACAACAG |

**TABLE S4. Primers used in this study**
